# Monthly Summary

**Published:** 1874-11

**Authors:** 


### MONTHLY SUMMARY.

*The Connection of Cancer and Skin Disease.*—Mr. George Gaskoin cites a number of cases in the London *Medical Times and Gazette*, to show the close relation between cancer and skin diseases. Thus, a boy with psoriasis has a sister with malignant disease; a man with psoriasis palmaris can give me no clue to his complaint beyond the fact that his grandfather had cancer. I have been inclined to believe that psoriasis lay closer to cancer than any other skin affeetion, but I am a little shaken in this opinion by what I have recently observed of acne in the children of the cancerous. Until lately I was disposed to look upon acne as a reverberation—a distant echo—of the cancer; but in many instances I find its succession close and direct. I have seen several cases of psoriasis where the tongue is affected as well as the cutaneous surface; many of these are not syphilitic. I cannot conceive that to such affections of the tongue one should deny the name of psoriasis; but no one case arises in my memory of epithelioma affecting the tongue where psoriasis was simultaneously displayed upon the skin, and the record of such a case is very desirable. It is now some years since I removed the tongue for an epithelioma supervening on a condition which I perfectly recollect, having observed every stage of its growth. The disease began in denuded patches, and there were hard portions, but not of that thickness I find recently described. These descriptions correspond very well to the horny and corn-like masses we observe in palmar psoriasis. Certainly it could never have entered into my mind to call such a condition ichthyosis. And here, as in the instance before mentioned, in spite of the advance in histology, I do not see marked out any broad and sharp line by which we can divorce cancer from its congeners, if I may so term these diseases of the skin. I will not go so far as to claim for cancer the character of a skin disease, but when we look at the variety as well as prodigality displayed in the expenditure of epithelium as seen in psoriasis, one cannot but here acknowledge so very close an affinity as to bind attention to the common character of each, both clinically and histologically. The affinity of lupus with psoriasis is well apparent in the clinical room; and this reminds me that certain forms of lupus in the extremities have been recently brought forward in Germany, which in the histological elements are scarcely, if at all, different from epithelioma.

As regards acne, I will say that in connection with it one will hear more of cancer than with any other disease that affects the skin; and the inquiry does not lead to epithelioma only, but to cancer in all its forms. I once thought that acne punctata gave far better fruit of inquiry than the other two; but both with acne simplex and acne rosacea (especially in women) I have found the parents to have been cancerous; I do not say very often, but sufficiently ofton to establish a firm connection. In some cases, all, or more than one, of the children have acne. Acne punctata is mostly accompanied with that sluggishness of temperament which some have identified with the cancerous diathesis.—*Med. and Surg. Reporter.*

---

*Gelseminum in Odontalgia.*—I desire to draw attention to the value of Gelseminum sempervirens in the treatment of some forms of odontalgia. Since reading Dr. Wickham Legg's paper, published in May last, advocating the employment of the drug in cases of odontalgia, I have frequently used the remedy for relief of toothache and some allied affections among my out-patients. Gelseminum, commonly called the yellow jasmine, is not very generally known to English practitioners, although it has been largely used in medicine for some years in the United States. The drug seems to act mainly upon the nervous system, impairing the sensibility of the sensory nerves. American pharmacists prepare a liquid extract; the dose of the powdered root is from one to two grains. I have used a tincture made from two ounces of coarsely-powdered gelseminum-root macerated in a pint of rectified spirit. In hospital out-patient practice, we meet with a large number of cases of neuralgia pains in the face and jaws, associated with carious teeth, but unconnected with any evident local inflammatory changes. The patients are frequently badly-nourished women. In such cases I have given the tincture of gelseminum in doses of fifteen minims every six hours in an ounce of dill-water. Out of about twenty cases, I do not think the use of the remedy has failed to be followed by decided and lasting relief in more than three or four instances. The pain did not usually disappear till after the third or fourth dose. I have seen enough of the employment of gelseminum to feel sure that more extended experience and careful investigation of its action will establish the drug as a valuable addition to our materia medica.—*Dental Cosmos.*

---

*How to Pull Teeth.*—The following, sent us by a reader, we respectfully refer to our Dental exchanges:—

A peculiar dental operation has just come under our observation. A certain citizen had an upper tooth which was loose and troublesome, so he resolved to extract it by fastening a string to it; but after a trial, finding the operation pained, he hadn't the grit to grin and bear it. He thought if the tooth could be extracted by some sudden mode, the pain would be but transient; and, after mature deliberation, he hit upon an ingenious plan to jerk it out in a jiffy. Procuring a heavy flat-iron, he tied it to the other end of the cord attached to his tooth, then shutting both eyes he let the iron "drop," which descended plumb centre on his pet corn. After hopping about the room, wildly, on one foot, groaning for very anguish of spirit, and reciting choice passages from profane history, he finally calmed down sufficiently to hurl the flat-iron over the fence, and swathe his sore toe in camphor and cotton. But, he pulled the tooth, and with it a piece of gum the size of a beefsteak. And the man lived.—*Med. and Surg. Reporter.*

---

*Oleate of Mercury in Treatment of Ringworm.*—Dr. Kane (*London Lancet*) describes several cases in which he had used the oleate of mercury in treating ringworm. He says it has the following advantages over other remedies:

1. It is a *certain remedy*, if carefully applied.
2. It *produces no staining* or injury of the skin. This is of great importance where the disease appears in the face.
3. It is *painless* in its application, which is not true of the strong parasiticides.
4. It *readily penetrates* the sebaceous glands, hair-follicles, and even into hairs themselves. Thus it is more likely to destroy the fungus than spirituous or aqueous solutions of mercury.

In very sensitive skins the irritation sometimes produced by it may be avoided by reducing the strength of the solution, and appplying it with a camel's-hair brush. The ordinary strength of the oleate is ten per cent.—*Nashville Journal of Medicine & Surgery.*

---

*A Lost Case.*—A physician in Vienna, Wis., who recently sued a man for services rendered in the treatment of his son for fracture of the thigh bone, lost his case. The local paper says that after the plaintiff had proven his case and rested, counsel for the defendant moved for a nonsuit, under chapter 95 laws of 1867, which in effect provides that no person practising physic and surgery shall be allowed to collect fees for services rendered unless he is a graduate of some medical college, or is a member of the State or some county medical society. The lawyer claimed that the plaintiff's duty was to prove he was a graduate, or was a member of one of the societies mentioned, and inasmuch as he had not done so, the defendant was entitled to a nonsuit. The motion was sharply resisted by the counsel for the plaintiff, but the defence sustained the theory by authorities, and the Justice dismissed the case.—*Med. Record.*

---

*To Suppress Hæmorrhage in the Mouth During Operations.*—Professor Andrews, of Chicago, (Med. Examiner,) advises when hæmorrhage interferes with operations in the mouth, to use the atomizer with ether, after etherization by inhalation, if the latter should be deemed necessary. He says that it does not interfere with the operation, that it restrains bleeding, and that the inhalation of the vapor, from the spray, keeps up a sufficient degree of anæsthesia.—*Medical Weekly.*

---

*The Medical Education of Women.*—Miss Philomene Ratinckx, of Antwerp, has passed a brillant examination in the anatomical sciences before the combined jury of Ghent and Louvain for the faculty of medicine. She has also obtained the royal diploma for the treatment of clubfoot. Two Circassian ladies at the Russian Academy of Medicine and Surgery, propose to return to their own country to practice the profession when they have taken their degrees.—*London Medical News.*

---

*Longevity of Medical Men.*—According to Casper the average age of clergymen is 65; of merchants , 62 ; clerks and farmers, 61 ; military men, 59; lawyers, 58; artists, 57; and medical men, 56. The medium duration of life in Russia he states at about 21 years; in Prussia, 29; in Switzerland, 34; in France, 35; in Belgium, 36; and in England, 38 years. It has been calculated by some statisticians that, under ordinary circumstances, men can live six or seven times longer than the years required to attain puberty. Taking this at the fourteenth year, from 84 to 98 years of age would be the natural life. The *Medical Times and Gazette* lately argued that medical men in England stand high on the scale of longevity. The united ages of twenty-eight physicians who died last year amount to 2,354 years, giving an average of more than 84 years to each. The youngest of the number was 80, the oldest 93 ; two others were 92 and 89 respectively; three were 87, and four were 86 each, Sir Henry Holland being one of the latter. There were also more than fifty whose average age was between 74 and 75 years. —*Doctor.*

---

*Cremation.*—According to *The Lancet* cremation makes progress in Germany, eighty-two cities now possessing cremation societies. The society at Berlin has received intelligence that the new furnace invented for the combustion of bodies by Professors Reclam and Siemens was tested on the 3d of June, and completely satisfied its constructors. Two hundredweights of animal carcass were consumed in about an hour and a half—reduced to white ashes, at no more than three shillings outlay. During the process no sound nor smell was appreciable. Solving as it does the question of cremation, Messrs Reclam and Siemens' contrivance has just met with a rival in the device of Dr. Steinbeis, President of the Central Institute for Trade and Industry, Wurtemberg. He proposes that the corpse should be placed in a trough made of cement, and be filled up with liquid cement so as completely to cover the dead body. The cement soon hardens and absorbs all the moisture from the body—converts it, in fact into a preservable mummy at small expense and trouble. As soon as the cement hardens, the square coffins may be piled one upon the other like blocks of stone, and the corpse is literally interred *in* instead of under its gravestone.—*Med. Record.*

---

*Table-fork swallowed by a Crazy Man; five or six years afterwards the Patient committed Suicide, when the Fork was found in his Stomach.*—A case of this kind had been communicated to the *Union Medicale*, April 9, 1874, by Dr. Baillarger.

A second case, communicated by M. Lascols, is published in the same Journal for 11th April. The subject of it was a married woman who in a fit of anger left her husband to go to her mother. When alone in her chamber she secretly swallowed a table-fork, a servant was charged with having stolen it, and the lady, though requesting that the servant should not be accused of the theft, did not reveal what she had done, and she lived as usual, not suffering much pain. After some months, however, the fork caused her pain and produced an enlargement of her stomach. A consultation of physicians was held, and M. Quairoche made an incision into the stomach and withdrew the fork, which had become perfectly black. The patient recovered and subsequently had children. Several similar cases are referred to in *Gaz. Hebdom*, for April 10, 1874, p. 228-9—one recorded as early as the year 1716.

---

*The Danger of Mixing Chromic Acid with Glycerine*—In preparing mixtures of this kind, which have been so highly recommended for diseases of the mouth and throat, care should be taken not to add these reagents together too quickly, for it is said that the fluid may take fire and explode. There is no danger if the glycerine be added drop by drop, for then a little warmth only will be generated.—*Il Raccogl. Med., Memorabilieu*—*Med. Record.*

